# Prediction of apolipoprotein A-I and high-density lipoprotein cholesterol in the neurological impairment and relapse of neuromyelitis optica spectrum disorder

**DOI:** 10.3389/fnins.2025.1629357

**Published:** 2025-07-15

**Authors:** Yanyan Wang, Feng Wang, Teng Huang, Ziling Zeng, Li Jiao, Hao Sun, Xiaoyu Zhang, Baojie Wang, Rujia Liu, Shougang Guo

**Affiliations:** ^1^Department of Neurology, Shandong Provincial Hospital, Shandong University, Jinan, China; ^2^Department of Neurology, Rongcheng People's Hospital Affiliated to Jining Medical University, Weihai, China; ^3^Department of Neurology, Shandong Second Provincial General Hospital, Jinan, China; ^4^Department of Neurology, Shandong Provincial Hospital Affiliated to Shandong First Medical University, Jinan, China; ^5^Department of Neurology, Liaocheng People's Hospital, Liaocheng, China; ^6^Department of Neurology, Yantai Yuhuangding Hospital Affiliated to Qingdao University Medical College, YanTai, China

**Keywords:** NMOSD, ApoA-I, HDL-C, neurological impairment, relapse

## Abstract

**Background and purpose:**

Neuromyelitis Optica Spectrum Disorder (NMOSD) is a rare inflammatory demyelinating disorder of the central nervous system, frequently resulting in irreversible neurological deficits such as blindness and paralysis. Emerging evidence suggests that dyslipidemia is associated with increased disability and poorer outcomes in autoimmune diseases. The purpose of the study is to investigate the associations between lipid profile with neurological impairment and relapse.

**Methods:**

This study retrospectively collected data from 130 hospitalized patients with AQP4-IgG positive NMOSD. Based on the Expanded Disability Status Scale (EDSS) scores at admission, patients were categorized into a mild-to-moderate group (EDSS ≤ 5.5) and a severe group (EDSS ≥ 6). All included patients were followed for at least 1 year, and were further divided into relapse and non-relapse groups based on whether a relapse occurred during the follow-up period. Logistic regression analysis was used to identify independent risk factors associated with the severity of neurological impairment and relapse. Receiver operating characteristic (ROC) curve analysis was conducted to determine the cutoff value of apolipoprotein A-I (ApoA-I) in predicting severe neurological impairment. Spearman correlation analysis was performed to assess the relationships among ApoA-I, high-density lipoprotein cholesterol (HDL-C) and CRP.

**Results:**

Multivariate binary logistic regression analysis revealed that ApoA-I (OR = 0.138, 95% CI: 0.021–0.902, *p* = 0.039) and the number of spinal cord lesion segments (OR = 1.368, 95% CI: 1.181–1.584, *p* < 0.001) were independent risk factors for the severity of neurological impairment. The area under the ROC curve (AUC) for ApoA-I in predicting the severity of neurological impairment was 0.647 (95% CI: 0.542–0.751), with a cutoff value of 1.165, a sensitivity of 59.4%, and a specificity of 67.6%. Multivariate logistic regression analysis identified HDL-C (OR = 0.082, 95% CI: 0.008–0.847, *p* = 0.036), clinical phenotype—specifically, compared to optic neuritis, myelitis (OR = 0.130, 95% CI: 0.037–0.458, *p* = 0.002), brainstem/cerebral syndrome (OR = 0.070, 95% CI: 0.007–0.731, *p* = 0.026), and mixed phenotypes (OR = 0.107, 95% CI: 0.018–0.642, *p* = 0.014) —as well as the use of subsequent monoclonal antibody therapy (OR = 0.190, 95% CI: 0.045–0.799, *p* = 0.023) as independent protective factors against relapse. Spearman correlation analysis showed that ApoA-I and HDL-C were significantly negatively correlated with CRP (*r* = −0.230, *p* = 0.008; *r* = −0.310, *p* < 0.001, respectively).

**Conclusion:**

Reduced levels of ApoA-I and HDL-C were associated with more severe neurological deficits and an increased risk of relapse.

## 1 Introduction

Neuromyelitis optica spectrum disorder (NMOSD) is a rare inflammatory demyelinating disease of the central nervous system (CNS) that primarily affects the optic nerves and spinal cord, often resulting in substantial and frequently permanent neurological deficits and disabilities, including blindness and paralysis (Wingerchuk and Lucchinetti, 2022). NMOSD has been reported worldwide with a prevalence of 0.7 to 10.0 per 100,000 (Wingerchuk and Lucchinetti, 2022). East Asians appear to have a higher prevalence of NMOSD (around 3.5 per 100,000) as compared to the Whites and other Asian racial groups, and the prevalence among the Chinese population is 3.31 per 100,000 (Hor et al., 2020). Pathogenic aquaporin-4 immunoglobulin G (AQP4-IgG) is identified in the serum of over 80% of NMOSD patients (Lucchinetti et al., 2014). AQP4-IgG in peripheral serum crosses the impaired blood–brain barrier (BBB) into the CNS, binds to AQP4 on astrocytic end-feet, and triggers the classical complement cascade ultimately causing oligodendrocyte damage and demyelinating injury (Jarius et al., 2020). Seronegative NMOSD represents a heterogeneous group of disorders with mechanisms that remain poorly understood, and is recognized as a relatively benign disease course (Yeo et al., 2019; Dauby et al., 2022). More than 90% of patients experience relapses, and each relapse can lead to progressive accumulation of disability (Wingerchuk and Lucchinetti, 2022). Among untreated AQP4-IgG positive patients, nearly one-quarter require gait assistance within 5 years of disease onset, more than 40% experience blindness in at least one eye, and the mortality rate approaches 10%, posing a significant disease burden on both patients and their families (Kitley et al., 2012; Ajmera et al., 2018). Therefore, early assessment of disability status and relapse risk at the time of NMOSD diagnosis is crucial for long-term disease management and evaluation of pharmacological treatment outcomes.

Lipids are fundamental components of cellular membranes and play crucial roles in energy supply, signal transduction, and other physiological functions. In the CNS, the myelin sheath is a lipid-rich multilamellar structure formed by oligodendrocytes (Stadelmann et al., 2019). Tian et al. (2020) conducted the first nationwide survey covering all 1,665 tertiary hospitals and revealed that approximately 12.8–13.9% of Chinese NMOSD patients are comorbid with hyperlipidemia. An animal study revealed that genetic deletion of ApoA-I in mice resulted in lymphocyte activation and autoantibody production, suggesting ApoA-I maybe a critical regulator of immune homeostasis (Wilhelm et al., 2009). A research suggested that dyslipidemia with low HDL-C is associated with disease activity of NMOSD (Cho et al., 2020). HDL modulates immune homeostasis through fine-tuning cholesterol bioavailability in lipid rafts, thereby influencing the activation states of B cells and T cells (Norata et al., 2012). Given the correlation between lipid profiles and autoimmune diseases, as well as the link between lipid metabolism and immune homeostasis, we hypothesized that lipid profiles may correlate with neurological impairment and relapse in NMOSD. Therefore, the present study aims to investigate the associations of lipid profiles with disability and relapse, and to evaluate the potential predictive value of lipid parameters for neurological deficit and prognosis of AQP4-IgG positive NMOSD.

## 2 Materials and methods

### 2.1 Participants

This study retrospectively enrolled patients admitted to the Department of Neurology at Shandong Provincial Hospital from August 2016 to September 2022. All patients were in the acute phase upon admission and fulfilled the diagnostic criteria of 2015 International Panel for NMO Diagnosis (IPND) (Wingerchuk et al., 2015). Patients were confirmed to be AQP4-IgG positive based on cell-based assay (CBA) testing. The exclusion criteria for this study were as follows: (1) AQP4-IgG seronegativity or unknown AQP4-IgG status; (2) non-first episode; (3) coexisting autoimmune diseases, including thyroiditis, systemic lupus erythematosus, Sjögren’s syndrome, or other autoimmune conditions; or comorbidities affecting lipid metabolism, such as hypertension, diabetes, nephrotic syndrome, or hypothyroidism; (4) use of lipid-lowering agents or corticosteroids prior to admission within 30 days pre-admission, or initiation of lipid-lowering therapy during follow-up; (5) incomplete clinical data or loss to follow-up.

### 2.2 Data collection

We retrospectively collected demographic and clinical data of eligible patients, including gender, age at onset, prodromal symptoms, serum AQP4-IgG titers, type of attack (optic neuritis, myelitis, brainstem/cerebral syndrome, or mixed lesions), Expanded Disability Status Scale (EDSS) scores, lipid profiles, coexisting autoantibodies, complete blood counts, biochemical profiles, and thyroid function tests. The lipid profile encompassed measurements of triglycerides (TG), total cholesterol (TC), high-density lipoprotein cholesterol (HDL-C), low-density lipoprotein cholesterol (LDL-C), lipoprotein (a) [Lp(a)], apolipoprotein A-I (ApoA-I), and apolipoprotein B (ApoB). Coexisting autoantibodies include antinuclear antibody profile, vasculitis antibody panel and thyroid-related antibodies. Additionally, magnetic resonance imaging (MRI) findings, treatments administered during the acute and subsequent phases, and maintenance therapies were recorded.

### 2.3 Clinical definition

EDSS was assessed by neurology specialists and used to evaluate the degree of neurological impairment in NMOSD patients. According to the preivious study, patients with an EDSS score≤5.5 were categorized into the mild-to-moderate group (MMG), while those with a score≥6.0 were assigned to the severe group (SG) (Wei et al., 2024). All eligible patients were followed for 12 months. Relapse was defined as either the worsening of pre-existing symptoms or the appearance of new symptoms lasting at least 24 h, with the clinical presentation meeting the 2015 diagnostic criteria by IPND.

### 2.4 Statistical analysis

Statistical analyses were conducted using IBM SPSS Statistics version 27. Continuous variables with a normal distribution were compared using the independent samples t-test, while those not normally distributed were analyzed using the Mann–Whitney U test. Categorical variables were assessed using the chi-square test. Binary logistic regression analysis was performed to identify independent risk factors associated with the severity of neurological impairment and relapse. Receiver operating characteristic (ROC) curve analysis was employed to evaluate the predictive value of ApoA-I for severe neurological impairment. Spearman correlation analysis was used to explore the associations between ApoA-I, HDL-C, and CRP levels.

A two-tailed *p*-value of <0.05 was considered statistically significant.

## 3 Results

### 3.1 Demographic and clinical characteristics of MMG and SG

A total of 130 patients were enrolled in this study, with 86.92% (*n* = 113) being female and a mean age of 44.71 ± 13.49 years (Supplementary Table S1). There were significant differences between the two groups in age at first onset, initial clinical manifestations, and the number of spinal cord segments involved (*p* < 0.05). Levels of HDL-C and ApoA-I were significantly higher in the MMG compared with the SG (*p* = 0.030, *p* < 0.001, respectively) (Table 1).

**Table 1:** Demographic and clinical characteristics of patients with different EDSS score.

Variables	MMG (*n* = 96)	SG (*n* = 34)	*p* value
Gender (female) *n* (%)	85 (88.54)	28 (82.35)	0.533
Age at onset, years	42.98 ± 13.19	49.59 ± 13.32	0.014
BMI (kg/㎡)	23.07 (21.52, 24.98)	23.07 (21.52, 24.98)	0.676
Prodromal symptoms *n* (%)	10 (10.42)	8 (23.53)	0.107
Serum AQP4-IgG titers *n* (%)			0.835
≥1:100	50 (52.08)	17 (50.00)	
<1:100	46 (47.92)	17 (50.00)	
Clinical phenotype n(%)			0.008
Optic neuritis	37 (38.54)	3 (8.83)	
Myelitis	35 (36.46)	21 (61.76)	
Brainstem/cerebral syndrome	11 (11.46)	3 (8.82)	
Mixed	13 (13.54)	7 (20.59)	
NLR	2.44 (1.88, 3.47)	2.95 (2.01, 4.16)	0.078
TG (mmol/L)	1.09 (0.74, 1.59)	1.27 (0.90, 1.68)	0.246
TC (mmol/L)	5.24 (4.42, 5.97)	5.03 (4.41, 6.02)	0.539
HDL-C (mmol/L)	1.53 ± 0.43	1.36 ± 0.34	0.030
LDL-C (mmol/L)	3.01 (2.54, 3.65)	3.10 (2.45, 3.65)	0.943
Lp(a) (g/L)	0.12 (0.06, 0.21)	0.15 (0.06, 0.19)	0.985
ApoA-I (g/L)	1.35 (1.22, 1.47)	1.08 (0.83, 1.16)	<0.001
ApoB (g/L)	0.98 (0.78, 1.17)	0.94 (0.83, 1.18)	0.998
Coexisting autoantibodies *n* (%)			0.543
≥2	45 (46.88)	18 (52.94)	
<2	51 (53.12)	16 (47.06)	
Cr (μmol/L)	48.00 (43.08, 55.00)	46.10 (39.55, 50.58)	0.090
UA (μmol/L)	209.50 (171.00, 258.75)	191.50 (166.00, 253.50)	0.283
CysC (mg/L)	0.91 (0.78, 1.08)	0.89 (0.71, 1.10)	0.647
CRP (mg/L)	0.82 (0.41, 1.71)	0.56 (0.25, 2.00)	0.357
FT3 (pmol/L)	3.40 (2.99, 3.82)	3.14 (2.40, 3.75)	0.087
FT4 (pmol/L)	15.65 (12.60, 18.17)	14.68 (13.46, 16.94)	0.781
TSH (μIU/mL)	0.81 (0.42, 1.44)	0.78 (0.39, 1.57)	0.958
C1q (mg/L)	180.40 ± 37.17	181.29 ± 34.68	0.903
Spinal cord leison segments *n* (%)	3 (0, 5)	2 (0, 4)	0.421

Numerical data are mean ± SD, median (IQR), *n* (%). MMG (mild-to-moderate group) was defined EDSS score≤5.5; SG (severe group) was defined EDSS score≥6. BMI, body mass index; Mixed, Simultaneously combining two or more subclinical phenotypes together; NLR, neutrophil-to-lymphocyte ratio; Cr, creatinine; UA, uric acid; Cys C, Cystatin C; CRP, C reactive protein; FT3, free triiodothyronine; FT4, free thyroxine; TSH, thyroid stimulating hormone.

### 3.2 Factors associated with severity of neurological impairment of AQP4-IgG positive NMOSD at disease onset

Univariate logistic regression analysis demonstrated that age at onset (OR = 1.039, 95% CI: 1.007–1.071, *p* = 0.017) and the number of spinal cord segments involved (OR = 1.371, 95% CI: 1.190–1.580, *p* < 0.001) were positively associated with severity of neurological impairment. In contrast, optic neuritis (OR = 0.132, 95% CI: 0.030–0.573, *p* = 0.007), HDL-C (OR = 0.310, 95% CI: 0.106–0.907, *p* = 0.033), ApoA-I (OR = 0.115, 95% CI: 0.022–0.612, *p* = 0.011), and FT3 (OR = 0.584, 95% CI: 0.342–0.998, *p* = 0.049) were negatively associated with EDSS scores.

Multicollinearity analysis was conducted on lipid parameters, including TG, TC, HDL-C, LDL-C, ApoA-I, and ApoB. All variance inflation factor (VIF) values were less than 10, indicating no significant multicollinearity among the lipid profile variables.

Variables with a *p*-value < 0.1 in the univariate analysis, including age at onset, prodromal symptoms, clinical phenotype, HDL-C, ApoA-I, FT3 (free triiodothyronine), and the number of spinal cord lesion segments, were included in the multivariate logistic regression analysis. After adjusting for potential confounders such as gender, age, and BMI, multivariate binary logistic regression revealed that ApoA-I (OR = 0.138, 95% CI: 0.021–0.902, *p* = 0.039) and the number of spinal cord lesion segments (OR = 1.368, 95% CI: 1.181–1.584, *p* < 0.001) were independent risk factors associated with the severity of neurological impairment of NMOSD (Table 2).

**Table 2:** Logistic regression analysis of factors for neurological impairment severity.

Variables	Univariate analysis			Multivariate analysis		
	OR	95%CI	*p* value	OR	95%CI	*p* value
Gender						
Female	0.604	(0.205–1.783)	0.361	–		–
Male	Refer			–		–
Age at onset	1.039	(1.007–1.071)	0.017	1.030	(0.993–1.069)	0.113
BMI	0.987	(0.844–1.154)	0.868	–		–
Prodromal symptoms						
Yes	2.646	(0.947–7.397)	0.064	2.674	(0.716–9.981)	0.143
No	Refer			Refer		
Serum AQP4-IgG titers						
<1:100	1.087	(0.497–2.377)	0.835	–		–
≥1:100	Refer			–		–
Clinical phenotype						
Optic neuritis	0.132	(0.030–0.573)	0.007	0.609	(0.104–3.566)	0.582
Myelitis	0.975	(0.347–2.741)	0.962	1.177	(0.322–4.309)	0.805
Brainstem/cerebral syndrome	0.295	(0.052–1.692)	0.171	1.461	(0.196–10.907)	0.712
Mixed	Refer			Refer		
NLR	1.155	(0.948–1.407)	0.152	–		–
TG	1.099	(0.632–1.912)	0.737	–		–
TC	0.932	(0.694–1.250)	0.637	–		–
HDL-C	0.31	(0.106–0.907)	0.033	0.684	(0.077–6.114)	0.734
LDL-C	1.001	(0.655–1.531)	0.996	–		–
Lp(a)	0.662	(0.100–4.377)	0.668	–		–
ApoA-I	0.115	(0.022–0.612)	0.011	0.138	(0.021–0.902)	0.039
ApoB	1.205	(0.385–3.776)	0.749	–		–
Coexisting autoantibodies						
<2	0.784	(0.358–1.717)	0.543	–		–
≥2	Refer			–		–
FT3	0.584	(0.342–0.998)	0.049	0.630	(0.322–1.234)	0.178
FT4	0.968	(0.867–1.082)	0.569	–		–
TSH	0.962	(0.739–1.251)	0.771	–		–
C1q	1.001	(0.990–1.012)	0.902	–		–
Spinal cord leison segments	1.371	(1.190–1.580)	<0.001	1.368	(1.181–1.584)	<0.001

EDSS was used to evaluated the neurological impairment severity. EDSS score≤5.5 was defined mild-to-moderate group (MMG), and score≥6.0 was assigned to the severe group (SG).

### 3.3 ROC curve of ApoA-I predicts the severity of neurological impairment at disease onset of AQP4-IgG positive NMOSD

**Figure 1:**
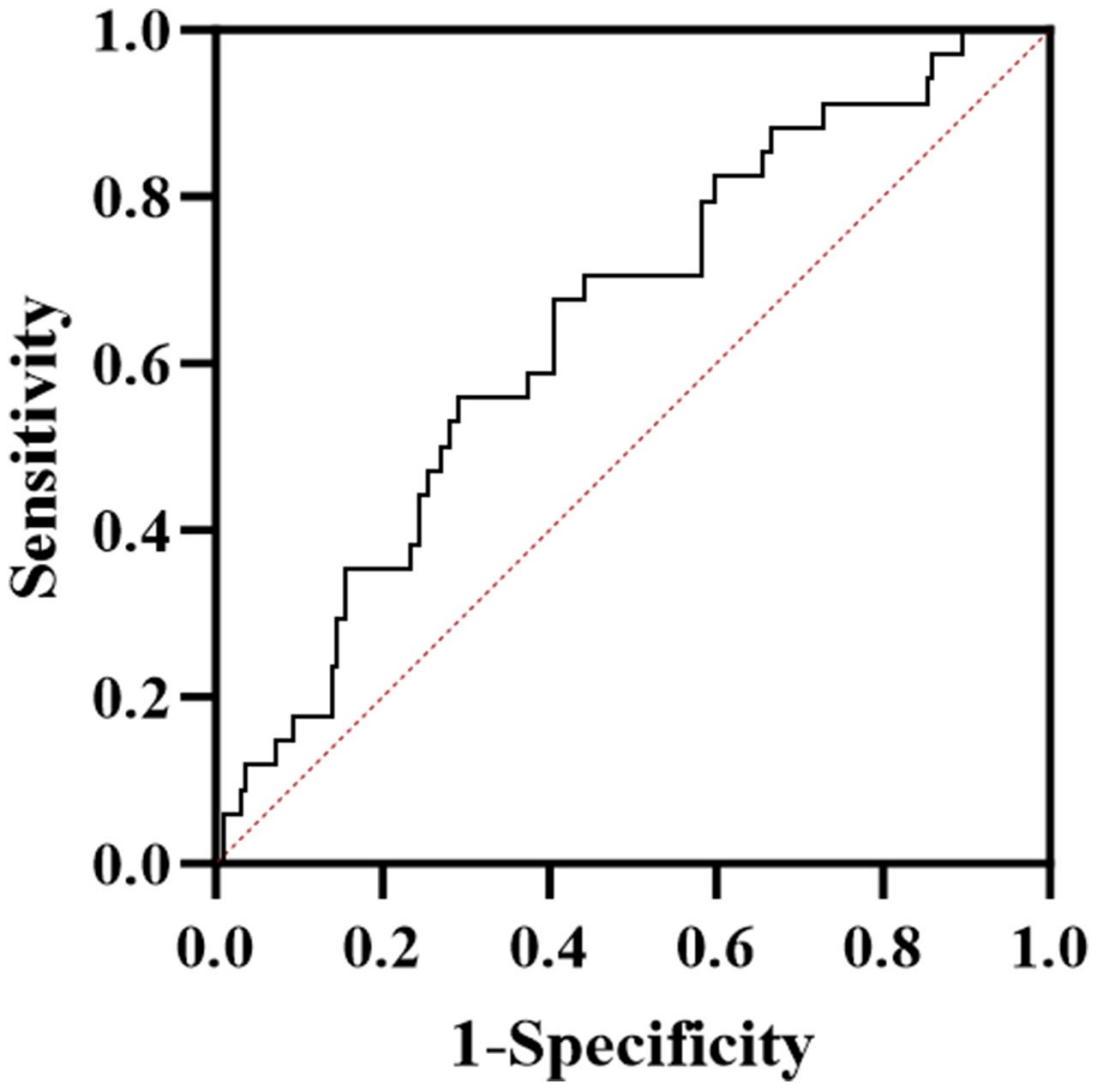
ROC curve analysis of the predictive power of ApoA-I for neurological deficit severity in NMOSD patients.

The ROC curve analysis demonstrated that ApoA-I could predict the severity of neurological impairment at the first attack in patients with AQP4-IgG positive NMOSD (Figure 1). The area under the curve (AUC) was 0.647 (95% CI: 0.542–0.751), with an optimal cutoff value of 1.165, achieving a sensitivity of 59.4% and a specificity of 67.6% (Table 3).

**Table 3:** ROC analysis of ApoA-I predicting the degree of neurological impairment.

AUC	SE	95%CI	Cut-off value	Sensitivity	Specificity
0.647	0.053	0.542–0.751	1.165	0.594	0.676

### 3.4 Demographic and clinical characteristics of relapse group and non-relapse group

In the relapse group, 82.61% of patients were female, with a mean age at onset of 48.57 ± 9.69 years, whereas in the non-relapse group, 87.85% were female, with a mean age at onset of 43.88 ± 14.07 years. Significant differences were observed between the two groups in terms of initial clinical manifestations and the use of sequential monoclonal antibody agents (*p* = 0.012 and *p* = 0.047, respectively). While HDL-C levels were lower in the relapse group compared to the non-relapse group, and Lp(a) and ApoA levels were comparable between the two groups, these differences were not statistically significant (Table 4).

**Table 4:** Demographic and clinical characteristics of patients in the relapse and non-relapse group.

Variables	Relapse (*n* = 23)	Non-relapse (*n* = 107)	*p* value
Gender (female) *n* (%)	19 (82.61)	94 (87.85)	0.737
Age at onset, years	48.57 ± 9.69	43.88 ± 14.07	0.061
BMI (kg/㎡)	23.01 (20.81, 25.39)	23.05 (21.48, 24.27)	0.942
Prodromal symptoms *n* (%)	4 (17.39)	15 (14.02)	0.928
Clinical phenotype *n* (%)			0.012
Optic neuritis	14 (60.87)	26 (24.30)	
Myelitis	6 (26.09)	50 (46.73)	
Brainstem/cerebral syndrome	1 (4.34)	13 (12.15)	
Mixed	2 (8.70)	18 (16.82)	
EDSS score	3 (3, 5)	3 (2, 6)	0.609
NLR	2.31 (2.02, 3.66)	2.63 (1.91, 3.81)	0.568
TG (mmol/L)	1.39 (0.81, 2.08)	1.11 (0.76, 1.56)	0.152
TC (mmol/L)	5.77 ± 1.86	5.15 ± 1.21	0.141
HDL-C (mmol/L)	1.39 (1.06, 1.63)	1.46 (1.23, 1.81)	0.174
LDL-C (mmol/L)	3.23 (2.68, 4.04)	3.00 (2.44, 3.51)	0.085
Lp(a) (g/L)	0.12 (0.60, 0.24)	0.12 (0.06, 0.20)	0.698
ApoA-I (g/L)	1.18 (0.92, 1.40)	1.18 (1.05, 1.0.42)	0.968
ApoB (g/L)	1.04 (0.89, 1.34)	0.94 (0.78, 1.15)	0.062
Coexisting autoantibodies *n* (%)			0.324
≥2	9	54	
<2	14	53	
CysC (mg/L)	0.91 (0.78, 1.0.13)	0.90 (0.76, 1.08)	0.683
CRP (mg/L)	0.70 (0.30, 1.69)	0.75 (0.39, 1.90)	0.498
FT3 (pmol/L)	3.54 (2.95, 4.22)	3.36 (2.89, 3.71)	0.286
FT4 (pmol/L)	16.01 (12.60, 17.25)	15.28 (12.90, 18.20)	0.896
TSH (μIU/mL)	1.01 (0.57, 1.62)	0.72 (0.40, 1.49)	0.135
C1q (mg/L)	187.11 ± 42.04	179.24 ± 35.14	0.349
Spinal cord leison segments	0 (0, 4)	3 (0, 5)	0.230
Acute phase treatment *n* (%)			0.427
IVMP	15 (65.21)	69 (64.48)	
IVMP+IVIG	6 (26.09)	34 (31.78)	
IVMP+PE	2 (8.70)	4 (3.74)	
Subsequent monoclonal antibody agents *n* (%)			0.047
Yes	4 (17.39)	42 (39.25)	
No	19 (82.61)	65 (60.75)	
Maintenance therapy *n* (%)			0.974
Prednisone	12 (52.17)	55 (51.40)	
Prednisone+MMF	9 (39.13)	41 (38.32)	
Prednisone+AZA	2 (8.70)	11 (10.28)	

IVMP, intravenous methyl prednisolone; IVIG, intravenous mmunoglobulin; PE, plasma exchange; MMF, mycophenolate mofetil; AZA, azathioprine.

### 3.5 Factors associated with relapse of AQP4-IgG positive NMOSD

Taking relapse within 1 year after the initial onset as the dependent variable, variables with *p* < 0.1 in the univariate analysis — including clinical phenotype, TC, TG, HDL-C, LDL-C, ApoB, and subsequent monoclonal antibody therapy — were incorporated in the multivariate logistic regression analysis. After adjusting for confounding factors such as sex, BMI, and AQP4 antibody titer, the multivariate logistic regression analysis showed that HDL-C, clinical phenotype, and subsequent monoclonal antibody agents were independent risk factors for relapse in AQP4-IgG + NMOSD (Table 5).

**Table 5:** Logistic regression analysis of risk factors for relapse.

Variables	Univariate analysis			Multivariate analysis		
	OR	95%CI	*p* value	OR	95%CI	*p* value
Gender						
Female	0.657	(0.193–2.235)	0.501	–		–
Male	Refer			–		–
Age at onset	1.026	(0.992–1.062)	0.133	–		–
BMI	1.052	(0.883–1.253)	0.57	–		–
Serum AQP4-IgG titers						
<1:100	1.198	(0.486–2.952)	0.695	–		–
≥1:100	Refer			–		–
EDSS score	1.008	(0.833–1.219)	0.939	–		–
Clinical phenotype						
Optic neuritis	Refer			Refer		
Myelitis	0.223	(0.077–0.648)	0.006	0.130	(0.037–0.458)	0.002
Brainstem/cerebral syndrome	0.143	(0.017–1.208)	0.074	0.070	(0.007–0.731)	0.026
Mixed	0.206	(0.042–1.021)	0.053	0.107	(0.018–0.642)	0.014
NLR	0.924	(0.696–1.225)	0.581	–		–
TG	1.713	(0.950–3.088)	0.073	1.223	(0.567–2.637)	0.608
TC	1.369	(0.992–1.888)	0.056	1.821	(0.552–6.005)	0.325
HDL-C	0.342	(0.100–1.167)	0.087	0.082	(0.008–0.847)	0.036
LDL-C	1.634	(1.022–2.612)	0.040	1.842	(0.621,5.462)	0.271
Lp(a)	2.342	(0.423,12.983)	0.330	–		–
ApoA-I	0.745	(0.144–3.861)	0.726	–		–
ApoB	4.047	(1.176–4.922)	0.027	0.047	(0.009–5.697)	0.212
CRP	0.960	(0.827–1.114)	0.593	–		–
FT3	1.253	(0.724–2.166)	0.420	–		–
FT4	0.977	(0.860–1.109)	0.718	–		–
TSH	1.115	(0.877–1.417)	0.376	–		–
C1q	1.006	(0.994–1.018)	0.347	–		–
Spinal cord leison segments	0.984	(0.865–1.119)	0.802	–		–
Subsequent monoclonal antibody agents						
Yes	0.241	(0.068–0.864)	0.029	0.190	(0.045–0.799)	0.023
No	Refer			Refer		

### 3.6 Correlation analysis of ApoA-I, HDL-C and CRP of NMOSD patients

**Figure 2:**
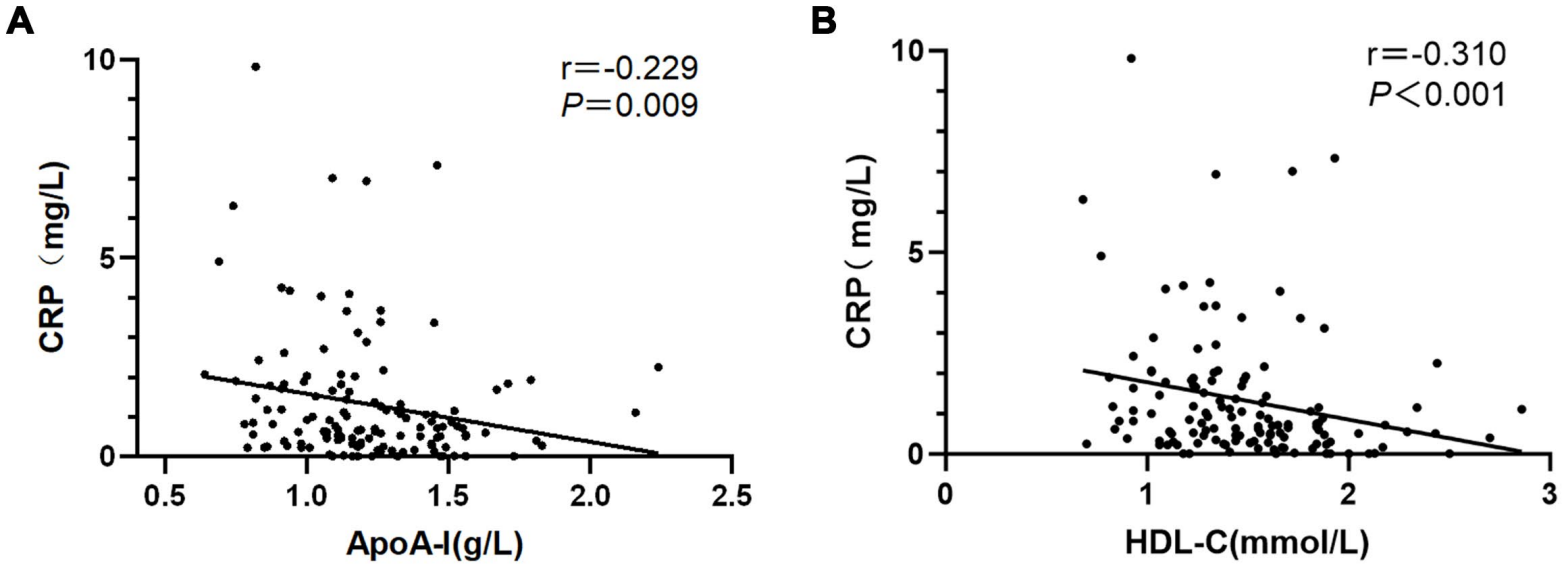
Associations between **(A)** ApoA-I and **(B)** HDL-c with CRP in NMOSD.

Spearman correlation analysis revealed a negative correlation between ApoA-I and CRP, with a correlation coefficient of −0.230 and a significant difference (*p* = 0.008). Similarly, HDL-C was negatively correlated with CRP, with a correlation coefficient of −0.310, which was also statistically significant (*p* < 0.001) (Figure 2).

## 4 Discussion

In this retrospective study, we investigated correlations between lipid parameters and neurological deficits as well as relapses. The results found that lower ApoA-I and HDL-C levels were associated with greater neurological impairment and higher relapse risk of NMOSD. Therefore, ApoA-I and HDL-C may serve as potential biomarkers for acute-phase disability assessment and prognosis evaluation.

Emerging evidence has been reported to evaluate the degree of neurological impairment and relapse in NMOSD. Although serum GFAP has been proposed as a reliable indicator of disease severity and activity in AQP4-IgG positive patients (Schindler et al., 2021; Kim et al., 2022), its clinical application remains limited due to low specificity and the burden of high cost. Thus, more practical and economic biomarkers in disease assessment of NMOSD are needed. Latest study found that high ApoA-I and HDLwere associated with relapse and attack of NMOSD (Li et al., 2025). The divergence between studies likely arised from distinct lipid measures: prior work analyzed lipoprotein concentrations (HDL/LDL), while our study specifically evaluated cholesterol carried by these lipoproteins (HDL-C/LDL-C), which may represent different pathophysiological aspects. Furthermore, proteomic analysis in this study revealed that the differentially expressed proteins (DEP)were associated with inflammatory response and lipid metabolism (Li et al., 2025). This result was consistent with the correlations observed between ApoA-I, HDL-C, and CRP in our study. Ding et al. demonstrated a positive association between elevated LDL-C and relapse risk (Ding et al., 2024). The study findings also indicated that cholesterol metabolism was significantly associated with relapse in NMOSD. However, this study found an association between elevated LDL-C and increased relapse risk, contrasting with our results which indicated reduced HDL-C was linked to higher relapse risk. This discrepancy may arise from differences in the enrolled populations: this study cohort was recruited from Southern China, whereas our participants were from Northern China, suggesting potential regional variations. Prior studies suggested that environmental factors may contribute to the pathogenesis of NMOSD, although conclusive evidence remains elusive (Wingerchuk and Lucchinetti, 2022). Previous studies also demonstrated associations between the lipid profile with disease activity and prognosis in NMOSD, with HDL-C being significantly lower during attacks than in remission, and triglycerides showing positive correlations with neurological disability severity and poor prognosis (Wu et al., 2019; Cho et al., 2020). ApoA-I was associated with neuroaxonal injury and cerebral perfusion of multiple sclerosis (MS) (Jakimovski et al., 2020; McComb et al., 2020). HDL-C exerts protective effects against BBB injury and also associated with cerebral perfusion of MS (Fellows et al., 2015; Jakimovski et al., 2020). The above studys revealed that dyslipidemia is closely related to the inflammation demyelinat on of CNS. Therefore, ApoA-I and HDL-C may be protective against the severity of neurological disability and relapse in NMOSD. Furthermore, we found that ApoA-I and HDL-C exhibit an inverse correlation with CRP via univariable linear regression. Given that this was an exploratory study rather than an in-depth investigation of mechanism, we did not adjust for potential confounders, and this finding is consistent with Li et al.’s proteomic discovery that DEP were enriched in chronic inflammatory response pathways (Li et al., 2025).

The mechanisms underlying the association between lipid metabolism and autoimmune diseases remain to be fully elucidated. Lipids, as fundamental structural components of cell membranes, not only establish selective barriers but also play pivotal roles in cellular signaling—either directly as signaling molecules or indirectly by modulating membrane fluidity (Mutlu et al., 2021). Dysregulated lipid metabolism can further aggravate autoimmune inflammation in addition to driving the development of atherosclerosis. Extracellular lipids not only provide metabolic energy and nutrient sources for B cells but also serve as key signaling mediators in their adaptive immune responses. ApoA-I primarily exerts its anti-inflammatory effects by modulating the functions of immune cells, including monocytes/macrophages, dendritic cells, neutrophils, and lymphocytes (Tao et al., 2024). A proteomic analysis of peripheral serum from patients with NMOSD revealed that ApoB and ApoC4 were up-regulated in the NMOSD group, and Gene Ontology enrichment analysis indicated that the DEP were involved in processes of inflammation response and lipids regulation, such as complement activation, chronic inflammation response, and the integrity of the BBB (Li et al., 2025). This quantitative proteomics analysis suggests that apolipoproteins may play a critical role in the pathological mechanisms of NMOSD. Another proteomic analysis of serum from AQP4-IgG positive NMOSD patients founded that DEP in the relapse and attack severity groups were mainly enriched in cytokine–cytokine receptor interaction, the IL-17 signaling pathway and the chemokine signaling pathway (Wei et al., 2024). A meta analysis of whole-genome sequences revealed that two independent signals were identified within the major histocompatibility complex (MHC) region associated with AQP4-IgG, one of which may be mediated by structural variation in the complement component 4 gene (Estrada et al., 2018). The study demonstrated that HDL-C correlated negatively with inflammatory cytokines, such as IL-6, IL-8, IL-10, in MS (Rádiková et al., 2020). Thus, lipid metabolism may exert pathogenic effects in NMOSD through multiple mechanisms, including cytokine pathways, complement activation, and compromised blood–brain barrier integrity.

The study possesses several strengths. First, it was designed with stringent inclusion and exclusion criteria to enhance data reliability. Given the pathological heterogeneity between AQP4-IgG positive and negative neuromyelitis optica spectrum disorder (NMOSD), only AQP4-IgG–positive patients were enrolled to ensure cohort homogeneity. Second, thyroperoxidase antibodies and other autoantibodies are detectable in up to half of AQP4-IgG-seropositive NMOSD patients, with approximately one-third exhibiting comorbid autoimmune disorders such as thyroiditis, systemic lupus erythematosus, or Sjögren’s syndrome (Wingerchuk and Lucchinetti, 2022). The study revealed that autoimmune thyroid disorders may contribute causally to NMOSD susceptibility (Wang et al., 2022). Therefore, we excluded patients with comorbid autoimmune disorders and adjusted for thyroid hormone levels in the analysis. Additionally, patients with potential confounding factors that could influence lipid metabolism, including the use of lipid-lowering agents, corticosteroid therapy (Martinez et al., 2024), or comorbidities such as hypertension, diabetes mellitus, or nephrotic syndrome were also excluded.

This study has several limitations that should be acknowledged. First, it was a single-center retrospective analysis with a relatively short follow-up duration, which may not fully capture long-term disease progression or lipid profile fluctuations. Lipid levels can be affected by external factors, including dietary habits and medication use. Given the retrospective nature of the study, it was hard to control all potential confounding variables. Second, as a retrospective study, cytokine and complement profiles were not assessed in the majority of early-phase patients; thus, correlation analyses between serum lipid profiles and cytokine levels/complement components could not be performed. Third, the relationship between ApoA-I, HDL-C with AQP4-IgG titers could not be specified owing to the different time at which the AQP4-IgG was sampled. In addition, AQP4-IgG titers were measured in some patients after corticosteroids therapy. Fourth, stringent inclusion/exclusion criteria (e.g., inclusion of AQP4-IgG seropositive patients, exclusion of those with comorbid autoimmune disorders and use of lipid-lowering agents) resulted in a relatively small sample size, diminishing statistical power. Therefore, future prospective, multicenter studies with larger cohorts and extended follow-up periods are necessary to confirm and expand upon these findings. Meanwhile, the correlations between serum lipid profiles and cytokine levels, complement components and AQP4-IgG titers warrant further investigation.

In conclusion, this study demonstrates that low ApoA-I and HDL-C were associated with increased neurological impariment and a higher risk of relapse in NMOSD patients with AQP4-IgG. Peripheral ApoA-I and HDL-C could serve as a potential, simple and accessible biomarkers in the early evaluation of disease severity and relapse risk in NMOSD, which may aid in optimizing acute-phase treatment strategies and facilitate risk stratification for long-term disease management.

## Data Availability

The original contributions presented in the study are included in the article/Supplementary material, further inquiries can be directed to the corresponding author.
