# Supplementary material for: Prediction of apolipoprotein A-I and high-density lipoprotein cholesterol in the neurological impairment and relapse of neuromyelitis optica spectrum disorder
Source: Front Neurosci. 2025 Jul 15;19:1629357. doi: 10.3389/fnins.2025.1629357 (PMC12303979; doi:10.3389/fnins.2025.1629357)
Supplement: Supplementary file 1 [file Table_1.DOCX]

Supplementary Table S1 Demographic and clinical characteristics of all enrolled patients.

| Variables | All(n＝130) |
| --- | --- |
| Gender(female) n(%) | 113(86.92) |
| Age at onset,years | 44.71±13.49 |
| BMI(kg/㎡) | 23.03（21.48,24.63） |
| Clinical phenotype n(%) |  |
| Optic neuritis | 40(31.77) |
| Myelitis | 56(43.08) |
| Brainstem/cerebral syndrome | 13(10) |
| Mixed | 21(16.15) |
| TG(mmol/L) | 1.14(0.77,1.60) |
| TC(mmol/L) | 5.18(4.43,5.99） |
| HDL-C(mmol/L) | 1.44（1.23,1.72） |
| LDL-C(mmol/L) | 3.05(2.53,3.64) |
| Lp(a)(g/L) | 0.12(0.06,0.20) |
| ApoA-I(g/L) | 1.18(1.03,1.41) |
| ApoB(g/L) | 0.95(0.79,1.16) |
| CRP(mg/L) | 0.75(0.33,1.73) |
| Severe group n(%) | 34(26.15) |
| Relapse n(%) | 23(17.69) |
